# Anti-Adhesion Effect of Composite Film Materials Based on Glycoluril-Modified Sodium Carboxymethyl Cellulose

**DOI:** 10.17691/stm2021.13.1.04

**Published:** 2021-02-28

**Authors:** A.A. Bakibaev, V.P. Tuguldurova, M.V. Lyapunova, V.V. Ivanov, O.A. Kaidash, E.V. Udut, M.V. Bukterov, E.E. Buyko, A.S. Kasyanova, V.S. Malkov

**Affiliations:** Engineer, Laboratory of Synthesis of High-Energy Compounds, Institute for Problems of Chemical and Energetic Technologies of the Siberian Branch of the Russian Academy of Sciences, 1 Sotsialisticheskaya St., Biysk, 659322, Russia; Engineer, Laboratory of Synthesis of High-Energy Compounds, Institute for Problems of Chemical and Energetic Technologies of the Siberian Branch of the Russian Academy of Sciences, 1 Sotsialisticheskaya St., Biysk, 659322, Russia; Engineer, Laboratory of Synthesis of High-Energy Compounds, Institute for Problems of Chemical and Energetic Technologies of the Siberian Branch of the Russian Academy of Sciences, 1 Sotsialisticheskaya St., Biysk, 659322, Russia; Associate Professor, Head of the Center for Preclinical Research, Central Research Laboratory, Siberian State Medical University, 2 Moskovsky trakt, Tomsk, 634050, Russia; Senior Researcher, Center for Preclinical Research, Central Research Laboratory, Siberian State Medical University, 2 Moskovsky trakt, Tomsk, 634050, Russia; Professor of the Russian Academy of Sciences, Head of Central Research Laboratory, Siberian State Medical University, 2 Moskovsky trakt, Tomsk, 634050, Russia; Researcher, Center for Preclinical Research, Central Research Laboratory, Siberian State Medical University, 2 Moskovsky trakt, Tomsk, 634050, Russia; PhD Student, Research School of Chemical and Biomedical Technologies, National Research Tomsk Polytechnic University, 30 Lenin Avenue, Tomsk, 634050, Russia, Researcher, Center for Preclinical Research, Central Research Laboratory, Siberian State Medical University, 2 Moskovsky trakt, Tomsk, 634050, Russia; Laboratory Assistant, Laboratory of Organic Synthesis, Research Office, National Research Tomsk State University, 36 Lenin Avenue, Tomsk, 634050, Russia; Head of the Laboratory of Organic Synthesis, Research Office, National Research Tomsk State University, 36 Lenin Avenue, Tomsk, 634050, Russia

**Keywords:** anti-adhesive agents, barrier films, composite materials, cytotoxicity of film materials, carbamide-containing heterocyclic compounds, glycoluril

## Abstract

**The aim of the study** was to develop composite film materials derived from modified sodium carboxymethyl cellulose and to evaluate their anti-adhesive effects.

**Materials and Methods.:**

The modified film materials were obtained by dissolving sodium carboxymethyl cellulose (Na-CMC) in an aqueous solution of a modifier (glycoluril) with subsequent homogenization and drying in a vacuum drying oven at room temperature. Physicomechanical parameters of the obtained films were determined using the Instron 3369 universal electromechanical testing machine (Great Britain) equipped with a climatic chamber (300–523 K), improved video extensometer, and the MKC-25 micrometer (Russia). Cytotoxicity of glycoluril-modified film materials derived from Na-CMC was studied by incubating cell cultures of 3T3-L1 mouse fibroblasts directly with extracts from films under study using a colorimetric test. Their anti-adhesion effect was investigated on 40 female Wistar rats by modeling a flat adhesion by inducing abrasion of the cecum and suturing the deserosed surface of the abdominal wall anatomically opposite the abrasion area. The presence of adhesions was assessed on day 8 after the operation. Commercial membrane Seprafilm (USA) was used as a reference sample.

**Results.:**

It was found that extracts obtained from film materials derived from Na-CMC modified with glycoluril at a concentration of 0.01 and 0.05 wt. % had no cytotoxic effect on the cell culture of mouse fibroblasts 3T3-L1. Flat adhesions were not detected when using Seprafilm. When film materials under study were placed in the abdominal cavity between the injured areas, formation of flat adhesions was not observed or observed in one case out of ten.

**Conclusion.:**

The obtained films are promising for preventing adhesions as a barrier-type agent. Modifying Na-CMC with glycoluril made it possible to create films that prevent formation of flat adhesions, have improved physical and mechanical properties and no cytotoxic effect.

## Introduction

Peritoneal adhesions develop in 64–100% of cases resulting from traumatic internal injuries, including those surgical. Their treatment is carried out surgically. However, preventive measures are taken already at the stage of surgical interventions on the abdominal organs. They involve creating a barrier around the internal organs in order to prevent formation of adhesions in the postoperative period [1]. However, optimal in terms of price and quality solution to this problem of abdominal surgery and gynecology has not been found yet.

At present, there are a number of barrier-type anti-adhesive agents [2] based on icodextrin [3, 4], polytetrafluoroethylene [5], hyaluronic acid [6], and cellulose [7], which are insufficiently effective [8] and have a number of limitations in application (low biodegradation rate [2], impossibility of use in peritonitis [9], etc.). In addition, anti-adhesive agents available on the domestic market (Interceed, Seprafilm, Adept, and others) are not produced in the Russian Federation.

Today, minimally invasive surgical techniques in combination with anti-adhesive barrier agents and various biocompatible substances are used to prevent adhesions in abdominal surgery. The most suitable film-forming material is sodium carboxymethyl cellulose (Na-CMC). It is a kind of polymeric cellulose ether obtained from natural cellulose by chemical treatment:

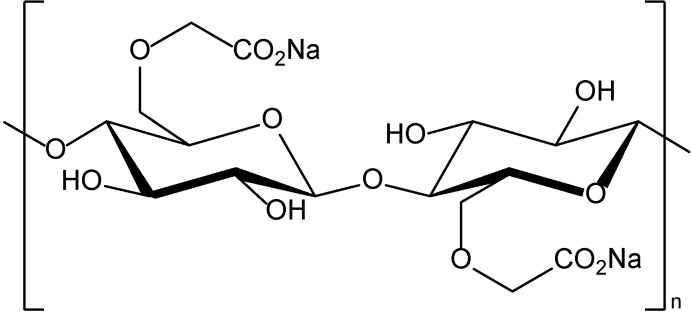



Na-CMC is readily soluble in water, forming a gel, it is non-toxic, non-carcinogenic, has no embryotoxic effect. Na-CMC is included in the State Register of Medicines of Russia under number 68/333/16 and in the Pharmacopoeia of the USA and Europe. Na-CMC has been mentioned in experimental works studying adhesion formation since the mid-80s [10]. It has been shown to be effective in combating adhesions when using 1–3% solutions, and a number of researchers note more significant anti-adhesion properties of Na-CMC as compared with other medicines of this series [11, 12].

At the same time, carbamide-containing heterocyclic compounds including glycoluril (2,4,6,8-tetraazabicyclo [3.3.0]octane-3,7-dione) and its derivatives are known to be used in pharmaceutical and cosmetic products. They have antibacterial and anti-inflammatory properties, stimulate tissue regeneration and prevent blood clots [13, 14]. Some derivatives of these compounds tend to polymerize and form films on the surface due to the large number of crosslinking functional groups. This combination of biological activity and chemical properties makes them promising components of anti-adhesion compounds.

**The aim of the study** was to develop composite film materials derived from modified sodium carboxymethyl cellulose and to evaluate efficacy of their anti-adhesion capacity.

## Materials and Methods

### Obtaining film materials.

Composite film materials were obtained from aqueous solutions of glycoluril of at least 99.0% purity and concentration of 0.01 and 0.05 wt. % using the following technology. At the first stage, 500 g of a 0.5% solution was prepared by introducing a portion (2.5 g) weighed on A&D HR-250AZG analytical balances (A&D Company, Japan) into a 500 ml calibrated flask, making the solution up to the mark with distilled water. Next, a 0.5% aqueous solution was diluted to a concentration of 0.01 and 0.05 wt. % in glass weighing bottles 34/12, 45 ml volume, to make a total of 40 g of each solution. The second stage involved adding the aqueous solution of glycoluril with the concentration of 0.01 or 0.05 wt. % to 0.2 g of Na-CMC film-forming compound with 250 kDa average molecular weight and 0.9 degree of substitution (Acros Organics, Belgium) to obtain a total of 10 g of solution. Homogenization was carried out for 30 min at room temperature (25°C) using the IKA RCT basic magnetic stirrer (IKA-Werke, Germany) equipped with a built-in thermoregulator. The solutions were placed into Petri dishes (100×20 mm according to GOST 23932-90) and dried for 8 h in the LT-VO/50 vacuum drying oven (Labteh, Russia) at a temperature not exceeding 25°C and residual pressure of no more than 50 mbar, using the Büchi V-710 vacuum pump equipped with a Büchi V-850 vacuum controller (Büchi AG, Switzerland).

The thickness of the obtained films was assessed using the MKC-25 micrometer (Russia) by measuring the average thickness at 5 points of the sample.

Tension tests of films were carried out using universal electromechanical machine Instron 3369 (Instron, UK) equipped with a climatic chamber (300–523 K) and an improved video extensometer. Samples under study had the form of a rectangle with 10 to 25 mm width and at least 150 mm length. Maximum sample width deviations did not exceed ±0.2 mm. The sample was placed in the grips, special attention being paid to the absence of distortions between the sample and the grips (to ensure deformation coaxiality), then stretching was performed (at least 3 repetitions for each sample). Measurement results were processed using Instron Bluehill software and displayed in coordinates of “time, load, elongation”, indicating the test conditions.

### Assessment of film material cytotoxicity.

Cytotoxicity of the samples was assessed in accordance with the requirements of GOST ISO 10993-5-2011 “Medical devices. Biological evaluation of medical devices. Part 5. Tests for *in vitro* cytotoxicity” by incubating cell cultures of mouse fibroblasts 3T3-L1 directly with extracts from the studied films using colorimetric analysis based on the endocytosis of *in vivo* neutral red dye, which is in positive correlation with the lysosomal function of living cells [15].

To prepare extracts, a film of 1.5×1.5 cm was placed under aseptic conditions in a pre-sterilized penicillin vial with 4 ml of complete culture medium (CCM — DMEM/F-12, 292 mg/L L-glutamine, 50 mg/L gentamicin, 10% FBS) and incubated at 37°C for 24 h (100% extract). The 50% and 25% extracts were obtained by sequential two-fold dilution of 100% extract in CCM.

After transferring cells to a 96-well plate in the quantity of 10,000 per well and reaching 80–90% confluence, the medium was removed, replaced with extracts (100 μl), and placed in a CO_2_ incubator for 24 h. An equivalent amount of CCM was added to the control cells. As a nonspecific positive control, 10 μl of 0.1% Triton X-100 detergent solution was added to the corresponding wells.

After incubation, the extracts were removed from the wells, the cells were washed once with 200 μl of 1X PBS, then 100 μl of CCM with neutral red (40 μg/ml) was added to each well. The plates were placed in a thermostat for 2 h at 37°C. Then the incubation medium with the dye was carefully removed, the cells were washed once with 200 μl of 1X PBS, and 150 μl of a mixture of 96% ethanol, deionized water, and glacial acetic acid (50:49:1) was added to each well to extract the bound dye. The optical density was measured on the Tecan Sunrise spectrophotometer (Tecan Group Ltd., Switzerland) at an operating wavelength of 540 nm (with a reference of 620 nm).

Cell viability was calculated as the ratio of living cells after incubation with extracts to living control cells, presented as a percentage of viable cells.

### Implantation of film materials.

The experiments were carried out on 40 female Wistar rats weighing 240±20 g. The experiments on animals were carried out at the Center for Preclinical Research of the Central Research Laboratory at the Siberian State Medical University (Tomsk, Russia) in full compliance with the international rules provided in “Guide for the Care and Use of Laboratory Animals” (National Research Council, 2011). Research protocols were drawn up in compliance with the European Convention for the Protection of Vertebrate Animals used for Experimental and Other Scientific Purposes (Strasbourg, 2006), and approved by the Ethics Committee of the Siberian State Medical University.

Surgical interventions on animals were performed under general anesthesia, including subcutaneous administration of 0.1% atropine sulfate solution (Moscow Endocrine Plant, Russia) at a dose of 0.2 mg/kg and intramuscular administration of Zoletil 100 (Virbac, France) at a dose of 50 mg/kg.

All animals underwent a sterile midline laparotomy (incision length of approximately 3 cm) and a flat adhesion was modeled by inducing abrasion of the visceral peritoneum on the cecum until bleeding was observed and deserosing of the left ventral wall with an area of 1 cm^2^ anatomically opposite the abrasion area of the cecum [16, 17]. To activate the adhesive process, two loops were sutured with monofilament polypropylene thread (LLC “Lintex”, Russia) on the deserosed surface of the abdominal wall. In animals of the control group (n=10), the injured cecum was placed back into the abdominal cavity near its injured wall without preliminary treatment. The obtained films (3.4_0.05_GUGK or 3.4_0.01_GUGK) or a reference sample (Seprafilm, USA) sized 1.5×1.5 cm were placed between the damaged surfaces in the animals of three experimental groups (n=10 in each). Isotonic sodium chloride solution in the amount of 2 ml was injected into the abdominal cavity and then it was sutured layer-by-layer with a continuous suture monofilament polypropylene thread (LLC “Lintex”) of conventional size (5/0). The wound surface was treated with an antiseptic and the animals were placed in individual cages. The total duration of the surgery was 50 min.

The experimental animals were sacrificed by CO_2_ asphyxiation on day 8 after modeling the flat adhesion. During necropsy, the abdominal cavity was opened, and the presence of adhesion between the abdominal wall and the cecum was visually assessed. The process was accompanied by photofixation.

### Statistical data processing.

*In vitro* data processing was performed using the GraphPad Prism7 software packages (GraphPad Software Inc., USA) and SPSS 17.0 (IBM, USA). Descriptive statistics was used for all data. The mean value and standard deviation were calculated. To assess normality of data distribution, Shapiro–Wilk test was used. One-way ANOVA was used for group comparison to determine significance of differences in multiple comparisons. Dunnett’s test was used for pairwise comparisons with control values. The differences were considered statistically significant at p<0.05.

## Results

The Table shows summary data regarding physical and mechanical properties of the obtained samples of composite film materials derived from Na-CMC modified with glycoluril, intended for biological research.

**Table:** Properties of composite film materials based on glycoluril-modified Na-CMC and Seprafilm commercial sample (USA)

Sample	Modifier	Modifier concentration (wt. %)	Average molecular weight of Na-CMC (kDa)	Degree of substitution with carboxyl groups in Na-CMC	Film thickness (mm)	Tension strength (MPa)	Young’s modulus (MPa)	Tensile stress-strain property (%)
3.4_0.01_GUGK	Glycoluril	0.01	250	0.9	0.05	46.40	2068.81	1.77
3.4_0.05_GUGK		0.05	250	0.9	0.03	40.58	2753.56	0.33
Seprafilm	—	—	—	—	0.05	32.02	2278.79	1.92

**Figure 1:**
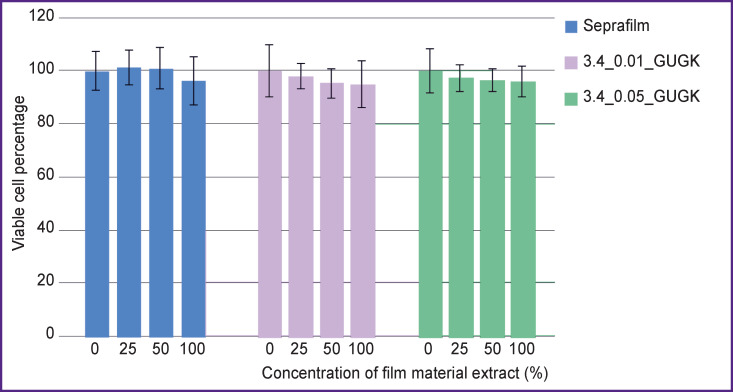
Influence of film materials modified with a carbamide-containing heterocyclic compound (glycoluril) and Seprafilm reference sample (USA) on viability of 3T3-L1 fibroblast cell culture; p<0.05 when compared to Seprafilm

It was found that extracts obtained from film materials 3.4_0.01_GUGK and 3.4_0.05_GUGK at concentrations of 100, 50, and 25% have no cytotoxic effect on cell cultures of mouse fibroblasts 3T3-L1 (Figure 1). The effect of the fabricated film materials on fibroblast cell viability was comparable to that produced by Seprafilm reference sample, which makes it possible to use them for assessment of flat adhesion prevention in experiments *in vivo*.

**Figure 2:**
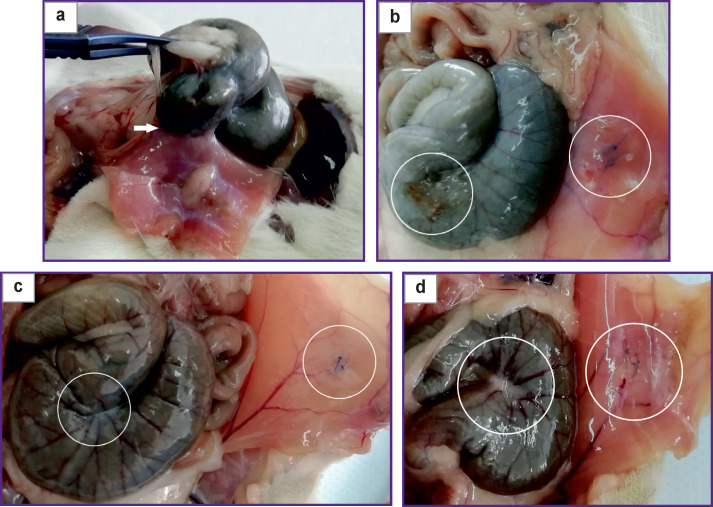
Photographs of the abdominal cavity on day 8 after modeling a flat adhesion between cecum abrasion area and the deserosed surface of the abdominal wall with sutures: (a) control (the arrow indicates a flat adhesion); (b) Seprafilm reference sample implantation; (c) 3.4_0.01_GUGK sample implantation; (d) 3.4_0.05_GUGK sample implantation; (b)–(d) the areas of cecum scarification and deserosed abdominal wall with sutures are circled

When testing the barrier-type anti-adhesion agent during *in vivo* experiments, there was detected adhesion process and presence of flat adhesions between the abrasion area of the cecum and the deserosed surface of the abdominal wall with sutured loops in 8 out of 10 animals of the control group (Figure 2 (a)). When using Seprafilm film (Figure 2 (b)), no flat adhesions were recorded in 100% of cases. When the investigated film 3.4_0.01_GUGK was placed in the abdominal cavity between the injured areas, formation of flat adhesions was not observed either. When using the 3.4_0.05_ GUGK film, adhesions formed in one case out of ten (Figure 2 (c), (d)).

## Discussion

Peritoneal adhesions remain one of the most difficult and still unsolved problems of abdominal surgery [2, 18, 19], which necessitates development and implementation of new adhesion prevention methods. One of the ways to reduce adhesions is to separate areas of the injured peritoneum, preventing their contact by means of membranes [2]. In recent decades, there is considerable research interest in the so-called anti-adhesion “barriers” made of Na-CMC-based materials, which are used to prevent formation of connective tissue between the injured organs. They create hydroflotation or separate the injured surfaces fixing on them and prevent formation of adhesions between them [20]. However, to achieve the anti-adhesion effect, Na-CMC is modified with various compounds [12, 21].

In our study, glycoluril, a carbamide-containing heterocyclic compound, was used as Na-CMC modifier, which made it possible to provide the target films with improved physical and mechanical properties and anti-adhesion effect comparable to that of Seprafilm reference film applied in clinical practice, including intra-peritoneal placement. Notably, our modification did not lead to cytotoxicity.

## Conclusion

Modifying Na-CMC with glycoluril has provided the possibility to create films 3.4_0.01_GUGK and 3.4_0.05_ GUGK that have suitable physical and mechanical properties, produce no cytotoxic effect, and revent formation of flat adhesions in the experimental modeling of adhesive process in the abdominal cavity. The obtained films are promising as a barrier-type agent for preventing adhesions.
